# Hierarchical Regression for Multiple Comparisons in a Case-Control Study of Occupational Risks for Lung Cancer

**DOI:** 10.1371/journal.pone.0038944

**Published:** 2012-06-11

**Authors:** Marine Corbin, Lorenzo Richiardi, Roel Vermeulen, Hans Kromhout, Franco Merletti, Susan Peters, Lorenzo Simonato, Kyle Steenland, Neil Pearce, Milena Maule

**Affiliations:** 1 Department of Medical Sciences, Cancer Epidemiology Unit, CeRMS and CPO-Piemonte, University of Turin, Turin, Italy; 2 Centre for Public Health Research, Massey University, Wellington, New Zealand; 3 Institute for Risk Assessment Sciences, Utrecht University, Utrecht, The Netherlands; 4 Department of Environmental Medicine and Public Health, University of Padua, Padua, Italy; 5 Rollins School of Public Health, Emory University, Atlanta, Georgia, United States of America; 6 Department of Medical Statistics, Faculty of Epidemiology and Public Health, London School of Hygiene and Tropical Medicine, London, United Kingdom; University of Bochum, Germany

## Abstract

**Background:**

Occupational studies often involve multiple comparisons and therefore suffer from false positive findings. Semi-Bayes adjustment methods have sometimes been used to address this issue. Hierarchical regression is a more general approach, including Semi-Bayes adjustment as a special case, that aims at improving the validity of standard maximum-likelihood estimates in the presence of multiple comparisons by incorporating similarities between the exposures of interest in a second-stage model.

**Methodology/Principal Findings:**

We re-analysed data from an occupational case-control study of lung cancer, applying hierarchical regression. In the second-stage model, we included the exposure to three known lung carcinogens (asbestos, chromium and silica) for each occupation, under the assumption that occupations entailing similar carcinogenic exposures are associated with similar risks of lung cancer. Hierarchical regression estimates had smaller confidence intervals than maximum-likelihood estimates. The shrinkage toward the null was stronger for extreme, less stable estimates (e.g., “specialised farmers”: maximum-likelihood OR: 3.44, 95%CI 0.90–13.17; hierarchical regression OR: 1.53, 95%CI 0.63–3.68). Unlike Semi-Bayes adjustment toward the global mean, hierarchical regression did not shrink all the ORs towards the null (e.g., “Metal smelting, converting and refining furnacemen”: maximum-likelihood OR: 1.07, Semi-Bayes OR: 1.06, hierarchical regression OR: 1.26).

**Conclusions/Significance:**

Hierarchical regression could be a valuable tool in occupational studies in which disease risk is estimated for a large amount of occupations when we have information available on the key carcinogenic exposures involved in each occupation. With the constant progress in exposure assessment methods in occupational settings and the availability of Job Exposure Matrices, it should become easier to apply this approach.

## Introduction

Occupational studies often involve the simultaneous analysis of multiple exposures and/or multiple occupations. A conventional approach to such analyses is to build a separate model for each occupation, adjusting for possible confounders. However, this approach treats all the associations equally, without accounting for the fact that some occupations are *a priori* more likely to be at risk than others, i.e. that some occupations have prior evidence of associations with the disease under study, whereas other occupations do not. Furthermore, for those occupations which show strongly elevated (or reduced) relative risks, their risk estimates may be biased away from the null due to random error, and it is likely that if the study were repeated, then risk estimates closer to the null would be found, due to ‘regression to the mean’.

Semi-Bayes adjustment methods have been shown to be valid approaches to these problems, particularly when the parameters to be estimated can be categorised into groups within which the various occupations or exposures have risks which are similar or “exchangeable” on the basis of *a priori* knowledge [1]. The basic idea of Semi-Bayes adjustment for multiple comparisons is that the observed variation of the estimated risks around their geometric mean will be larger than the variation of the true (but unknown) risks. The Semi-Bayes method [2] specifies an *a priori* value for the variation of the true risks; this *a priori* value is then used to adjust the observed risks [3]. The adjustment consists in shrinking outlying estimates towards the overall mean of the observed estimates. The larger the individual variance of the estimates, the stronger is the shrinkage, i.e. the shrinkage is stronger for less reliable estimates based on small numbers.

Semi-Bayes adjustment is a special case of the more general method of hierarchical regression [4]. The latter approach incorporates a number of specific types of regression model as special cases including Bayesian regression, Semi-Bayes regression, Stein regression, penalized likelihood regression, and ridge regression. In the current context, hierarchical regression can be used to incorporate prior similarities between the exposures of interest in a second-stage model. This approach has been used previously in several studies involving the assessment of multiple exposures/risk factors, e.g. studies on diet [5], genetic studies [6], [7] and occupational studies [8]–[10]. The objective of the present work was to re-analyse data from an occupational case-control study of lung cancer, applying hierarchical regression and including prior information from a validated Job-Exposure-Matrix (JEM). In particular, we included in the second-stage model the exposure to three known lung carcinogens for each occupation, under the assumption that occupations entailing similar exposure levels to the same lung carcinogen are associated with similar risks of lung cancer.

## Materials and Methods

### Ethics Statement

The current study is a re-analysis of the Italian subset of the multicentric study on lung cancer from the International Agency for Research on Cancer (IARC) [11], hence no additional ethical committee approval was requested.

### Description of the Data

The data are from a population-based case-control study conducted between 1990 and 1992 in two areas of Italy (the city of Turin and the Eastern part of Veneto Region). The study methodology has been described elsewhere [11]. Briefly, cases (956 men and 176 women) were all individuals diagnosed with incident primary lung cancer during 1990–1992, aged less than 75 and resident in the study areas. Controls (1,253 men and 300 women) were randomly selected from the local population registries and frequency matched with cases by gender, study area and five-year age groups. Information was collected on basic demographic details, active and passive smoking, and lifetime occupational history. In particular, the dates of beginning and ending work, as well as the job title and branch of industry, were recorded for each occupational period that lasted at least 6 months. Job titles and branches of industry were coded blind to case-control status according to the International Standard Classification of Occupations (ISCO-68) [12] and the International Standard Industrial Classification (ISIC) [13], respectively. The current analyses were carried out only in men.

We focused on three chemicals which were classified by the International Agency for Research on Cancer (IARC) [14] as group 1 carcinogens targeting the lung: asbestos, chromium and silica. Exposure to these carcinogens was assessed through a General Population Job Exposure Matrix (DOM-JEM) developed in 2010 by three occupational experts (HK, RV and SP) for a large pooled case-control study on lung cancer [15]. The DOM-JEM assigns an ordinal exposure score for several lung carcinogens (0=no exposure, 1=low exposure, 2=high exposure) to each ISCO code.

### Conventional Analysis

The analyses were done at the three-digit ISCO code level. For the ISCO codes starting by “X” (workers not classifiable by occupation) and for those specified to a maximum of 2 digits, all the corresponding occupational histories were deleted from the dataset, resulting in the exclusion of 5 cases and 14 controls. Only job-codes with at least ten subjects were retained in the analyses (n=129). The first-stage models estimated the risk of lung cancer for each of the 129 occupations separately. The Odds Ratio (OR) for ever being exposed to each job was modelled using unconditional logistic regression, adjusting for age, study area and cigarette smoking status (never, ex, current):
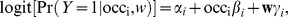
where *Y* is a dichotomous variable representing the lung cancer status (*Y*=1: cases; *Y*=0: controls), occ_i_ (i=1,…,129) is a dichotomous variable representing the exposure status to the i^th^ occupation, **w** is a vector of covariates included in the model (i.e. age, study area, and cigarette smoking status), 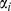 is the intercept term, 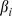 is the regression coefficient corresponding to the i^th^ occupation, and 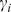 is the vector of regression coefficients corresponding to the covariates for the i^th^ occupation.

We also carried out conditional logistic regression. Since the estimates obtained through conditional and unconditional regression adjusting for matching variables were very similar, here we show only those obtained through unconditional logistic regression.

The ORs with corresponding 95% confidence intervals (CI) were estimated through maximum-likelihood using the SAS Logistic procedure.

### Hierarchical Regression

Hierarchical regression can be used to attempt to improve on maximum-likelihood (ML) estimates by using a second-stage linear model [5], [6]. The second-stage model used here regressed the ln(OR)s of the occupations on the occupations’ estimated exposure levels to asbestos, chromium and silica.
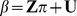


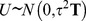
(2)

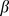
 is the 129-element vector of the ln(OR)s for the occupations. 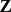
is the
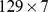
matrix (intercept and 2 indicator variables per exposure) obtained from the DOM-JEM [15] that classifies the 129 occupations according to their levels of exposure to asbestos, chromium and silica. Each carcinogen has two possible levels of exposure, expressed by two dichotomous variables.

More specifically, we have:


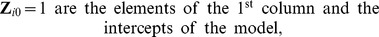
 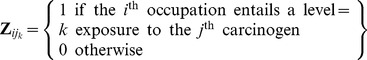
is the value at the 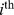 row and 
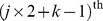
 column, where 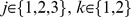, and 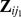
and 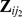
are mutually exclusive.

Appendix S1 shows rows 55 to 60 of matrix 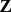. For example, 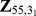
is located at the 55^th^ row and the 6^th^ column of the matrix and equals 1 because “nursery workers and gardeners” are exposed to silica (from soil) at level 1.


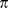
 is the 7-element vector (estimated by the second-stage model) of the coefficients corresponding to the effects on lung cancer of the levels of exposures to the three carcinogens described in 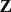.


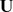
 is a 129-element vector of the error terms representing the residual effect of being employed in each occupation after accounting for the exposure to asbestos, chromium and silica.


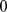
 is a 129-element vector of zeros.


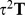
 is the 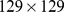 second-stage covariance matrix. The second-stage variance for an estimate for a particular occupation represents the residual variance of the effect of the occupation after taking into account the effects of the three lung carcinogens. This can be estimated from the data (Empirical Bayes) or specified *a priori* (Semi-Bayes). We used here the Semi-Bayes approach. 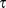 is a parameter used to control the strength of the common shrinkage of all the ML coefficients towards their prior means. We set 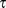 to 0.23, 0.41, 0.59 and 0.76, corresponding to the assumptions that 95% of relative risks would lie within a 2.5, 5, 10 and 20 fold-range of each other, respectively, if **T** was the identity matrix. We assumed that the second-stage variance for each occupation depends on its levels of exposure to the three carcinogens, so that the higher the levels of exposure, the smaller the second-stage variance. For ease of computation, 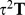 did not include residual correlation between occupations. 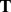 is then a diagonal matrix (see Appendix S2 for examples of calculation) with:
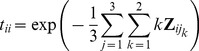
(3)


The model was fitted with R (free software for statistical computing and graphics) [16] (although such analyses can also be done in SAS and Stata, or with any logistic regression package by adding simple prior data [17]). The code is a modified version of the code provided by Chen and Witte [6] and is available on request. The coefficients 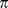 were estimated through weighted least squares (see Appendix S3). Substituting them back into the equation (2) yielded prior means 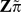 for the occupations’ coefficients 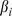. Hierarchical regression estimates (posterior estimates) for the coefficients for each occupation were then obtained by averaging the ML coefficients (from conventional analysis) and their respective prior means, so that the larger the diagonal elements of 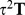, the stronger the shrinkage of coefficients towards their prior means.

### Semi-Bayes Adjustment towards the Global Mean

We compared the results obtained through hierarchical regression with those obtained through a more traditional Semi-Bayes adjustment towards the global mean, used previously in occupational studies involving multiple comparisons [3], [18]–[22]. The variance of the true ln(OR)s was assumed to be 0.25. Assuming a normal distribution of the ln(OR)s, this choice implies that the true ORs are within a 7-fold range of each other [2]. The Semi-Bayes adjustment was applied separately within two groups of occupations believed to entail different levels of exposure to lung carcinogens: the occupations held by white-collar workers (identified by the first digit of ISCO code<6, less likely to entail exposure to carcinogens) and the occupations held by blue-collar workers (identified by the first digit of ISCO code≥6, more likely to entail some or heavy exposure to carcinogens). For each group of occupations, this method was equivalent to a particular case of hierarchical regression in which only the intercept was included in the second-stage model.

## Results

Table 1 summarizes the basic characteristics of the subjects included in our analyses.

**Table 1:** Selected characteristics of cases and controls.

	Cases		Controls	
	N	(%)	N	(%)
***Center***				
Turin	482	(50.7)	669	(54.1)
Eastern Veneto region	469	(49.3)	568	(45.9)
***Age, years***				
**Mean, (Standard Deviation)**	62.3	(7.4)	63.3	(7.7)
***Cigarette smoking***				
Never smoker	15	(1.6)	248	(20.0)
Ex-smoker	327	(34.4)	587	(47.5)
Current smoker	609	(64.0)	402	(32.5)
**Total**	951		1237	

Table 2 presents the ORs of lung cancer for ever being exposed to each level of exposure of the carcinogens included in the second-stage model (asbestos, chromium and silica). These ORs were estimated through logistic regression models, adjusting for age, study area and cigarette smoking status (never, ex, current). Ever being exposed to each of the three carcinogens was associated with an increased risk of lung cancer, with higher risks observed for high levels of exposure.

**Table 2:** Odds ratio (OR) of lung cancer and 95% confidence intervals (CI) for ever being exposed to each level of exposure of asbestos, chromium and silica.

Carcinogen	Exposure level	Cases/Controls	OR[95%CI]^a^
**Asbestos**	**Unexposed (0)**	429/682	1.00
	**Ever low (1)**	477/512	1.43[1.18–1.73]
	**Ever high (2)**	45/43	1.62[1.01–2.61]
**Chromium**	**Unexposed (0)**	579/808	1.00
	**Ever low (1)**	270/339	1.11[0.90–1.37]
	**Ever high (2)**	102/90	1.55[1.11–2.15]
**Silica**	**Unexposed (0)**	627/862	1.00
	**Ever low (1)**	288/345	1.19[0.97–1.46]
	**Ever high (2)**	36/30	1.58[0.92–2.71]

a Estimated through logistic regression models, adjusting for age, study area and cigarette smoking status (never, ex, current).

Table 3 shows the descriptive statistics for the distribution of the 129 ln(OR)s obtained through ML estimation, Semi-Bayes (SB) adjustment, and Hierarchical Regression (HR) with 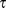
=0.76, 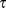
=0.59, 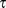
=0.41 and 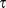
=0.23.

**Table 3:** Descriptive statistics for the distribution of the ln(OR)s of lung cancer for the 129 selected occupations (3-digit ISCO codes; n>10) obtained using Maximum Likelihood (ML), Semi-Bayes adjustment towards the global mean (SB) and hierarchical regression (HR).

	ML	SB	HR			
			*τ*=0.76	*τ*=0.59	*τ*=0.41	*τ*=0.23
**Maximum prior range of ORs’ variation**		7-fold range^a^	20-fold range^b^	10-fold range^b^	5-fold range^b^	2.5-fold range^b^
**Mean of estimated ln(OR)s**	−0.12	−0.07	−0.08	−0.07	−0.06	−0.04
**Median of estimated ln(OR)s**	−0.03	−0.06	−0.06	−0.07	−0.08	−0.08
**Standard deviation of estimated ln(OR)s**	0.63	0.31	0.41	0.35	0.28	0.20
**Mean of estimated standard errors**	0.45	0.32	0.37	0.34	0.28	0.20

a A 7-fold range means that we assume *a priori* that 95% of the true ORs are within a 7-fold range of each other (e.g. from 0.5 to 3.5).

b Prior range of ORs’ variation when matrix 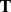
is the Identity matrix. A 20, 10, 5, or 2.5-fold range means that we have 95% *a priori* certainty that the residual OR for being ever employed in an occupation after accounting for the effect of the carcinogens will lie within a 20, 10, 5 or 2,5-fold range.

Compared with ML, the mean of the distribution of the ln(OR)s is pulled towards zero after SB and HR. For HR, this effect is stronger for smaller values of 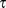. The standard deviation of the distribution of the ln(OR)s is also reduced by both SB and HR and is smaller for smaller values of 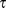 (Table 3). It can also be noted that both SB and HR estimates have on average smaller standard errors.

**Figure 1:**
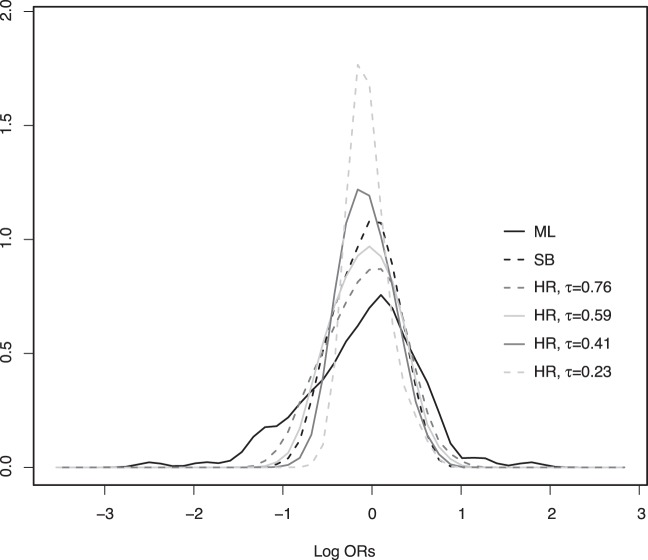
Kernel density distributions of the ln(OR)s. Kernel density distributions of the ln(OR)s of lung cancer for the 129 selected occupations obtained using Maximum Likelihood (ML), Semi-Bayes adjustment towards the global mean (SB) and hierarchical regression (HR).

The kernel density plots (Figure 1) of the ln(OR)s show less left skewed distributions for SB and HR than for the ML estimates (smaller medians after SB and HR are also apparent in Table 3). This is due to the fact that the extreme estimates, which are more likely to be unstable, are pulled towards their prior means.

In Table 3, we can see that, for SB, the mean and the standard deviation of the ln(OR)s distribution are included between the corresponding values for HR[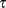
=0.41] and HR[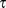
=0.59]. However, the distribution obtained after SB is more left skewed than after HR (Figure 1). The density curve for SB has a higher slope on its right side than on its left side: while the left side lies between the curves for HR[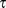
=0.41] and HR[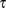
=0.59], the right side lies under both curves. This indicates that extreme positive estimates are in general shrunk more strongly towards the null value (ln(OR)=0) through SB than through HR.

**Figure 2:**
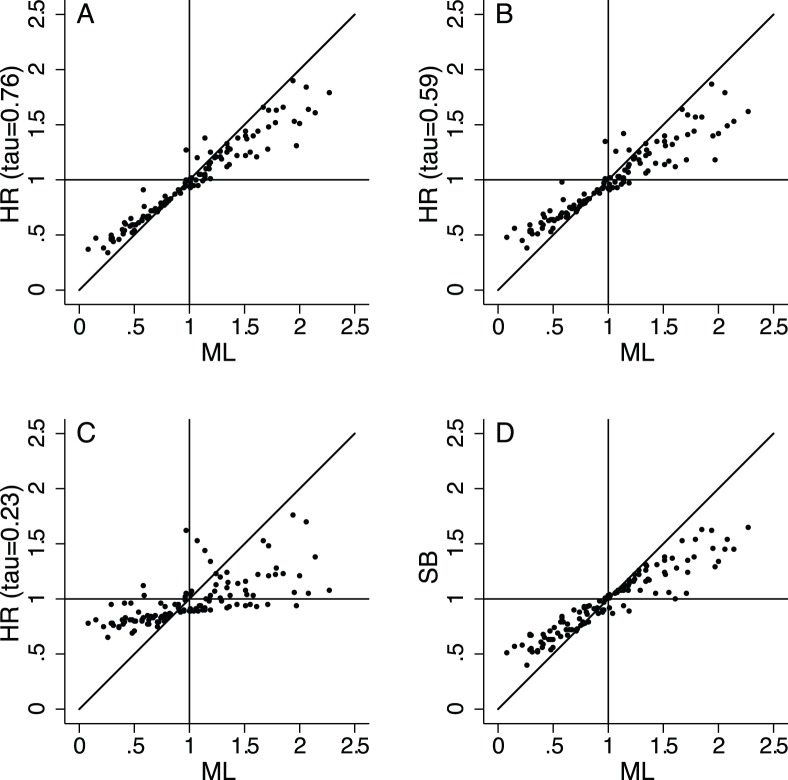
Relationship between the ORs obtained with the different approaches. Scatter plots of the ORs of lung cancer for the 129 selected occupations estimated using hierarchical regression (HR) with 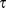
=0.76 *vs*. Maximum Likelihood (ML) (A), HR with 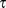
=0.59 *vs*. ML (B), HR with 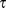
=0.23 *vs*. ML (C) and Semi-Bayes adjustment towards the global mean (SB) *vs.* ML (D).

The effect of the shrinkage can be seen in the scatter plots in Figure 2, where the ORs for each occupation estimated with HR and SB are plotted against ML estimates. The further ML estimates are from the null value (OR=1), the more scattered are HR and SB estimates and the stronger is the shrinkage. As expected, extreme estimates are pulled more strongly for smaller values of 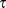.

Table 4 reports the OR estimates obtained through the different methods for the occupations associated with the twenty highest risks of lung cancer in the conventional analysis (ORs for all the occupations are available in Appendix S4). Shrinkage is particularly strong for specialised farmers (ML OR=3.44, SB OR=1.59, HR OR[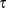
=0.76]=1.81, HR OR[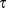
=0.23]=1.00) and for ships’ engine-room ratings, who are highly exposed to asbestos (ML OR=5.88, SB OR=1.54, HR OR[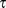
=0.76]=2.43, HR OR[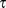
=0.23]=1.78). This is due to the fact that these two occupations are held by a small number of subjects and the confidence intervals for the ML estimates are therefore very large. Despite the large CIs, however, the ‘shrunk’ estimates still indicate that these occupations are associated with an increased risk of lung cancer, and their ORs are consistent with those of other occupations which involve exposure to lung carcinogens.

**Table 4:** ORs of lung cancer and 95% confidence intervals obtained using Maximum Likelihood (ML), Semi-Bayes adjustment towards the global mean (SB) and hierarchical regression (HR) for the occupations associated with the twenty highest ORs in the conventional ML analysis.

ISCO CODE - OCCUPATION	CASES/CONTROLS	ML	SB	HR				CARCINOGENIC EXPOSURE		
			*7-fold range*	*τ*=0.76 *(20-fold range)*	*τ*=0.59 *(10-fold range)*	*τ*=0.41 *(5-fold range)*	*τ*=0.23 *(2.5-fold range)*			
		OR[95%CI]	OR[95%CI]	OR[95%CI]	OR[95%CI]	OR[95%CI]	OR[95%CI]	ASB^a^	CR^a^	SI^a^
034-Electrical and electronics engineering technicians	5/9	1.61[0.44–5.88]	1.00[0.45–2.21]	1.21[0.45–3.22]	1.12[0.47–2.66]	1.02[0.51–2.02]	0.93[0.60–1.44]	0	0	0
628-Farm machinery operators	9/8	1.62[0.55–4.81]	1.28[0.62–2.65]	1.44[0.58–3.54]	1.38[0.59–3.22]	1.31[0.61–2.81]	1.22[0.62–2.40]	0	0	2
872-Welders and flame-cutters	47/37	1.67[1.03–2.71]	1.53[0.99–2.36]	1.66[1.06–2.59]	1.64[1.06–2.53]	1.60[1.07–2.41]	1.53[1.06–2.19]	0	2	0
039-Engineering technicians not elsewhere classified	7/7	1.71[0.51–5.72]	1.05[0.49–2.28]	1.28[0.50–3.28]	1.18[0.51–2.72]	1.05[0.54–2.07]	0.95[0.62–1.46]	0	0	0
727-Metal drawers and extruders	5/5	1.72[0.45–6.63]	1.24[0.56–2.76]	1.63[0.66–4.03]	1.59[0.72–3.52]	1.54[0.81–2.91]	1.48[0.94–2.33]	0	2	0
725-Metal moulders and coremakers	10/9	1.72[0.65–4.58]	1.35[0.67–2.70]	1.48[0.70–3.13]	1.41[0.72–2.76]	1.32[0.77–2.28]	1.21[0.83–1.77]	0	1	1
952-Reinforced-concreters, cement finishers and terrazzo workers	13/8	1.78[0.70–4.56]	1.38[0.70–2.74]	1.52[0.73–3.16]	1.44[0.75–2.78]	1.34[0.78–2.30]	1.22[0.84–1.77]	0	1	1
729-Metal processers not elsewhere classified	28/20	1.79[0.97–3.32]	1.54[0.91–2.61]	1.63[0.95–2.82]	1.57[0.94–2.62]	1.45[0.92–2.28]	1.28[0.91–1.80]	0	1	1
931-Painters, construction	42/29	1.85[1.09–3.15]	1.63[1.0–2.61]	1.66[1.0–2.71]	1.57[0.9–2.52]	1.43[0.9–2.20]	1.23[0.8–1.72]	1	0	0
871-Plumbers and pipe fitters	29/22	1.94[1.0–3.65]	1.62[0.9–2.77]	1.90[1.0–3.43]	1.87[1.0–3.32]	1.82[1.0–3.16]	1.76[1.0–2.98]	2	0	0
891-Glass formers, cutters, grinders and finishers	15/8	1.95[0.7–4.85]	1.46[0.7–2.87]	1.53[0.7–3.24]	1.40[0.7–2.78]	1.24[0.7–2.19]	1.06[0.7–1.57]	0	1	0
751-Fibre preparers	5/5	1.97[0.4–8.03]	1.29[0.5–2.89]	1.31[0.4–3.64]	1.18[0.4–2.90]	1.05[0.5–2.12]	0.94[0.6–1.46]	0	0	0
943-Non-metallic mineral product makers	7/4	2.00[0.5–7.13]	1.34[0.6–2.92]	1.51[0.6–3.56]	1.42[0.6–2.98]	1.31[0.7–2.35]	1.21[0.8–1.78]	0	1	1
723-Metal melters and reheaters	12/6	2.06[0.7–5.72]	1.45[0.7–2.96]	1.84[0.8–3.90]	1.79[0.9–3.50]	1.73[0.9–3.03]	1.70[1.1–2.61]	1	2	0
791-Tailors and dressmakers	14/11	2.08[0.8–5.00]	1.54[0.7–2.97]	1.64[0.7–3.50]	1.49[0.7–3.01]	1.28[0.7–2.32]	1.05[0.7–1.57]	0	0	0
893-Glass and ceramics kilnmen	11/6	2.14[0.7–6.25]	1.45[0.7–3.01]	1.61[0.7–3.42]	1.53[0.7–2.95]	1.44[0.8–2.45]	1.38[0.9–2.00]	1	1	1
796-Upholsterers and related workers	19/11	2.27[0.9–5.21]	1.65[0.8–3.13]	1.79[0.8–3.69]	1.62[0.8–3.18]	1.37[0.7–2.45]	1.08[0.7–1.62]	0	0	0
728-Metal platers and coaters	13/7	3.26[1.1–9.07]	1.81[0.8–3.72]	2.30[1.0–5.05]	2.08[1.0–4.23]	1.82[1.0–3.29]	1.57[1.0–2.46]	0	2	0
612-Specialised farmers	9/3	3.44[0.9–13.17]	1.59[0.7–3.55]	1.81[0.6–4.93]	1.53[0.6–3.68]	1.23[0.6–2.47]	1.00[0.6–1.55]	0	0	0
982-Ships’ engine-room ratings	8/2	5.88[0.9–36.71]	1.54[0.6–3.73]	2.43[0.7–7.46]	2.16[0.8–5.73]	1.93[0.8–4.29]	1.78[0.9–3.32]	2	0	0

a ASB=Asbestos (0=no exposure, 1=low exposure, 2=high exposure), CR=Chromium (0=no exposure, 1=low exposure, 2=high exposure), SI=Silica (0=no exposure, 1=low exposure, 2=high exposure).

SB with an *a priori* true standard deviation of 0.5 provided estimates that were less scattered than the HR estimates obtained with the chosen values of 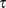 (Figure 2). In particular, SB shrunk all the increased ML estimates towards the null, whereas some increased estimates were pulled away from the null when using HR. For example, the ML risk estimate for “Metal smelting, converting and refining furnacemen” (ML OR=1.07, SB OR=1.06, HR OR[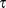
=0.59]=1.26, HR OR[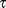
=0.41]=1.37) is close to the null whilst HR, weighting for their exposure to both asbestos (low exposure) and chromium (high exposure), pulls the risk estimate away from the null. Similarly, HR estimates a higher risk for “Miners and quarrymen” (ML OR=1.19, SB OR=1.14, HR OR[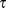
=0.59]=1.27, HR OR[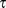
=0.41]=1.30), exposed to both asbestos (low exposure) and silica (high exposure). “Metal annealers, temperers and case-hardeners” (ML OR=1.14, SB OR=1.08, HR OR[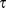
=0.59]=1.42, HR OR[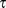
=0.41]=1.44) are only highly exposed to chromium and “Railway engine drivers and firemen” (ML OR=0.97, SB OR=1.01, HR OR[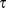
=0.59]=1.35, HR OR[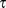
=0.41]=1.47) are only highly exposed to asbestos. However, the ML estimates have large variances, which increases the strength of the shrinkage towards the prior ORs and results in elevated risk estimates after HR. On the other side SB, using less informative priors, performs a more systematic shrinkage and results in a general reduction of the ORs. Some ML ORs below 1 are also shrunk above 1 by HR whereas they are shrunk upwards but below 1 by SB, as in the case of “Metal casters” (ML OR=0.58, SB OR=0.84, HR OR[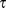
=0.76]=0.91, HR OR[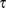
=0.59]=0.98, HR OR[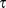
=0.41]=1.07, HR OR[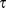
=0.23]=1.12). Therefore, in general, SB with an *a priori* true standard deviation of 0.5 and HR with 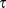
=0.59 provide shrinkages of similar magnitude, but different risk estimates for occupations known *a priori* to be exposed to lung carcinogens.

## Discussion

In our analyses, HR provided estimates which are likely to be more reliable and have narrower confidence intervals than are obtained with conventional ML analysis. Many of the more extreme estimates obtained through ML analysis are based on small numbers and have large confidence intervals. HR, by including prior information on exposure to three lung carcinogens in a second-stage model, pulls these estimates towards their respective prior means and thereby reduces their estimated standard errors and confidence intervals. The strength and direction of the shrinkage for the more extreme estimates depend on the prior estimated exposures of the corresponding occupations to the three carcinogens. For example, “specialised farmers” are not exposed to any of the considered carcinogens and HR therefore pulls the corresponding OR strongly towards the null value whereas the OR remains elevated for “metal melters and reheaters” who are exposed to both asbestos and chromium. In a situation of multiple comparisons, HR is thus a useful tool for data analysis which takes into account the multiple comparisons involved and the commonalities of exposures across different occupations.

In our analyses, HR and SB shrinkage had similar effects on the ML estimates. However, since HR uses more detailed prior information than SB, the shrinkage performed by the former method is likely to be more appropriate and specific than the latter (provided of course that this prior information is reasonably valid). Our findings show that all the estimates were shrunk towards the null value through SB whereas some of them were pulled in the opposite direction by HR, because of the use of additional prior information. Thus, both approaches aim at decreasing false-positive findings but HR also mitigates the inherent effect of the shrinkage of increasing false-negatives. On the other hand, SB is easier to compute and does not need the manipulation of a second-stage matrix. The choice between the two methods therefore essentially depends on the availability and the reliability of the information included in the second-stage model.

The HR shrinkage as proposed in this paper could have two relevant implications when conducting exploratory analyses on risks associated with occupations: i) it decreases the possibility that an occupation entailing exposure to important known occupational carcinogens is dismissed by the study, ii) it helps to pick up, among occupations not entailing exposure to known occupational carcinogens, those which should be further investigated and are more likely to provide information on the role of new or suspected occupational carcinogens. Our findings on construction painters, which were associated with an OR of 1.85 (95% CI: [1.0–3.15]) in the standard ML approach, are an example of the latter implication. According to the DOM-JEM construction painters are not exposed to chromium or silica and have a low exposure to asbestos. However, the OR remains elevated after HR even when using a 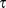 of 0.23 (OR=1.23, 95% CI: [0.8–1.72]), suggesting that any increased risk is due to other exposures. Thus it is worth conducting further studies on painters. Indeed a recent meta-analysis on 47 independent estimates of the association between employment as a painter and risk of lung cancer estimated an overall relative risk of 1.35 (95% CI: [1.2–1.41]), which is closer to our HR than our ML estimate [23]. If HR weighs information from the DOM-JEM too heavily, we might incur in the problem that high risks for occupations classified as unexposed to the 3 considered carcinogens (but likely to be exposed to other carcinogens) are always knocked down. Among the 20 occupations with the highest ML ORs, 6 were unexposed to asbestos, chromium or silica. HR shrinkage was strong for risks based on a small number of subjects, but did not nullify those based on larger numbers, such as upholsterers (ML OR: 2.27, HR OR [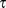
=0.59]: 1.62) and tailors/dressmakers (ML OR: 2.08, HR OR [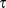
=0.59]: 1.49).

The inclusion of many covariates in the second-stage model can lead to collinearity problems and difficulties in estimating second-stage coefficients. For this reason, our analyses were restricted to three well known lung carcinogens from the DOM-JEM [15]. The JEM used here classifies the exposure to the carcinogens in three levels, and these were used to specify the second-stage model. Before fitting the model, we verified that a sufficient number of subjects were exposed to each level of the selected carcinogens to ensure model convergence. If this condition did not hold, a simpler version of the matrix with dichotomous exposure to the carcinogens could have been used. An interesting future development of this method could be the use of continuous exposure variables in the second-stage model.

In our analyses, we have assessed the impact of four different values of 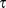. The choice of 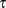 depends on how many second-stage covariates are included in the model, how strong and reliable their associations with both the outcome and the exposures of interest are, and how well the first-stage model was specified (i.e. if it can be assumed that all the relevant confounders have been included). In our analyses, we chose to include three well known strong occupational lung carcinogens, and our first-stage model was adjusted for smoking. It was therefore reasonable to assume that 95% of the estimates would lie within a maximum 10-fold-range of each other (e.g. between 0.5 and 5.0) after accounting for the second-stage covariates, and a 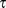 of 0.59 would then be appropriate. For each occupation, 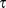 was inversely weighted by the amount of exposure to carcinogens as specified in the JEM. In this respect, HR is superior to SB since it modulates the weights given to the residual variation of each occupation and hence the amount of shrinkage towards prior information.

HR has already been shown to be a valid approach to adjust for multiple comparisons in studies involving the analysis of multiple occupational exposures and outcomes [10] and in occupational studies where the first-stage exposures (chemical and physical agents) were regressed on physicochemical properties in a second-stage model [8], [9]. In our analyses, we focused on the risks associated with the occupations and included carcinogens in a second-stage model. We found that HR could also be a valuable tool in occupational studies in which the risk of disease is estimated for a large amount of occupations when we have information available on the key carcinogenic exposures involved in each occupation. With the constant progress in exposure assessment methods in occupational settings and the construction and refinement of Job Exposure Matrices, it should become easier to have access to this information and carry out this type of analysis in the future.

## Supporting Information

Appendix S1Section of the matrix 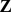
for six occupations (rows 55 to 60)(DOC)

Appendix S2Examples of calculation of the elements of the second-stage covariance matrix 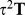

(DOC)

Appendix S3 Computation of the Hierarchical Regression estimates
(DOC)

Appendix S4 Odds Ratios of lung cancer and 95% confidence intervals obtained using Maximum Likelihood (ML), Semi-Bayes adjustment towards the global mean (SB) and hierarchical regression (HR) for the 129 selected occupations (3-digit ISCO codes; n>10)
(DOC)
