# Supplementary material for: Hierarchical Regression for Multiple Comparisons in a Case-Control Study of Occupational Risks for Lung Cancer
Source: PLoS One. 2012 Jun 11;7(6):e38944. doi: 10.1371/journal.pone.0038944 (PMC3372490; doi:10.1371/journal.pone.0038944)
Supplement: Appendix S1 — Section of the matrix for six occupations (rows 55 to 60) (DOC) [file pone.0038944.s001.doc]

**Appendix S1: Section of the matrix $Z$for six occupations (rows 55 to 60)**

| **Occupation** | **Z**’s **row** | **Elements of** $Z$ | | | | | | |
| --- | --- | --- | --- | --- | --- | --- | --- | --- |
|  |  | Intercept | Asbestos *low* (1) | Asbestos *high* (2) | Chromium *low* (1) | Chromium *high* (2) | Silica *low* (1) | Silica *high* (2) |
| 627-nursery workers and gardeners | 55 | 1 | 0 | 0 | 0 | 0 | 1 | 0 |
| 628-farm machinery operators | 56 | 1 | 0 | 0 | 0 | 0 | 0 | 1 |
| 631-loggers | 57 | 1 | 0 | 0 | 0 | 0 | 0 | 0 |
| 641-fishermen | 58 | 1 | 0 | 0 | 0 | 0 | 0 | 0 |
| 700-production supervisors  and general foremen | 59 | 1 | 0 | 0 | 0 | 0 | 0 | 0 |
| 711-miners and quarrymen | 60 | 1 | 1 | 0 | 0 | 0 | 0 | 1 |
