# Supplementary material for: Hierarchical Regression for Multiple Comparisons in a Case-Control Study of Occupational Risks for Lung Cancer
Source: PLoS One. 2012 Jun 11;7(6):e38944. doi: 10.1371/journal.pone.0038944 (PMC3372490; doi:10.1371/journal.pone.0038944)
Supplement: Appendix S2 — Examples of calculation of the elements of the second-stage covariance matrix (DOC) [file pone.0038944.s002.doc]

**Appendix S2: Examples of calculation of the elements of the second-stage covariance matrix** $\tau^{2}\mathbf{T}$

| **Occupation** | **Z**’s: ***i*th row** | **Carcinogenic exposure** | | | $t_{ii}$ | **Second-stage residual variance** $\tau^{2}t_{ii}$ | | | |
| --- | --- | --- | --- | --- | --- | --- | --- | --- | --- |
|  |  | ASB* | CR* | SI* |  | $\tau$=0.76 | $\tau$=0.59 | $\tau$=0.41 | $\tau$=0.23 |
| 627-nursery workers and gardeners | 55 | 0 | 0 | 1 | 0.72 | 0.42 | 0.25 | 0.12 | 0.04 |
| 628-farm machinery operators | 56 | 0 | 0 | 2 | 0.57 | 0.33 | 0.20 | 0.10 | 0.03 |
| 631-loggers | 57 | 0 | 0 | 0 | 1 | 0.58 | 0.35 | 0.17 | 0.05 |
| 641-fishermen | 58 | 0 | 0 | 0 | 1 | 0.58 | 0.35 | 0.17 | 0.05 |
| 700-production supervisors and general foremen | 59 | 0 | 0 | 0 | 1 | 0.58 | 0.35 | 0.17 | 0.05 |
| 711-miners and quarrymen | 60 | 1 | 0 | 2 | 0.37 | 0.21 | 0.13 | 0.06 | 0.02 |

*ASB=Asbestos(0 = no exposure, 1 = low exposure, 2 = high exposure)

CR=Chromium(0 = no exposure, 1 = low exposure, 2 = high exposure)

SI=Silica(0 = no exposure, 1 = low exposure, 2 = high exposure)
