# Supplementary material for: Hierarchical Regression for Multiple Comparisons in a Case-Control Study of Occupational Risks for Lung Cancer
Source: PLoS One. 2012 Jun 11;7(6):e38944. doi: 10.1371/journal.pone.0038944 (PMC3372490; doi:10.1371/journal.pone.0038944)
Supplement: Appendix S3 — Computation of the Hierarchical Regression estimates (DOC) [file pone.0038944.s003.doc]

**Appendix S3: Computation of the Hierarchical Regression estimates**

The second-stage coefficients $\pi$for the different categories of exposures to the three carcinogens are estimated through weighted least squares with

(A1)

where and $\hat{\mathbf{V}}$is a diagonal matrix composed of the estimated variances of the first stage coefficients estimated in the conventional analysis $\hat{\beta}$.

The Hierarchical Regression estimates $\tilde{\beta}$are then obtained by averaging the first stage coefficients $\hat{\beta}$ with their respective prior means $\mathbf{Z}\tilde{\pi}$

, where . (A2)

Their covariance matrix is estimated by where

*Example of calculation of the Hierarchical Regression estimates for the miners and quarrymen (ISCO: 711) (60^th^ row of $\mathbf{Z}$)*

|  |  | Maximum-likelihood | |  | Hierarchical regression | |
| --- | --- | --- | --- | --- | --- | --- |
|  | $\mathbf{Z}_{60}\times\tilde{\pi}$ | $\hat{\beta}_{60}$ | OR_60_ | $b_{ii}$* | $\tilde{\beta}_{60}$ | OR_60_ |
| $\tau$=0.76 | 0.31 | 0.17 | 1.19 | 0.39 | 0.22 | 1.25 |
| $\tau$=0.59 | 0.31 | 0.17 | 1.19 | 0.52 | 0.24 | 1.27 |
| $\tau$=0.41 | 0.31 | 0.17 | 1.19 | 0.70 | 0.27 | 1.30 |
| $\tau$=0.23 | 0.31 | 0.17 | 1.19 | 0.87 | 0.29 | 1.34 |

*diagonal elements of the matrix
