# Supplementary material for: Hierarchical Regression for Multiple Comparisons in a Case-Control Study of Occupational Risks for Lung Cancer
Source: PLoS One. 2012 Jun 11;7(6):e38944. doi: 10.1371/journal.pone.0038944 (PMC3372490; doi:10.1371/journal.pone.0038944)
Supplement: Appendix S4 — Odds Ratios of lung cancer and 95% confidence intervals obtained using Maximum Likelihood (ML), Semi-Bayes adjustment towards the global mean (SB) and hierarchical regression (HR) for the 129 selected occupations (3-digit ISCO codes; n>10) (DOC) [file pone.0038944.s004.doc]

**Appendix S4: Odds Ratios of lung cancer and 95% confidence intervals obtained using Maximum Likelihood (ML), Semi-Bayes adjustment towards the global mean (SB) and hierarchical regression (HR) for the 129 selected occupations (3-digit ISCO codes; n>10)**

| **ISCO code - Occupation** | **Cases/**  **Controls** | **ML** | **SB** *7-fold range* | **HR** | | | |
| --- | --- | --- | --- | --- | --- | --- | --- |
|  |  |  |  | ***τ*=0.76** *20-fold range* | ***τ*=0.59** *10-fold range* | ***τ*=0.41** *5-fold range* | ***τ*=0.23** *2,5-fold range* |
|  |  | **OR[95%CI]** | **OR[95%CI]** | **OR[95%CI]** | **OR[95%CI]** | **OR[95%CI]** | **OR[95%CI]** |
| 031-Surveyors | 4/17 | 0.31[0.10-0.98] | 0.52[0.24-1.12] | 0.44[0.18-1.12] | 0.51[0.22-1.16] | 0.61[0.31-1.19] | 0.76[0.50-1.17] |
| 032-Draughtsmen | 8/45 | 0.26[0.12-0.58] | 0.40[0.21-0.76] | 0.34[0.17-0.68] | 0.38[0.20-0.73] | 0.47[0.27-0.83] | 0.65[0.44-0.97] |
| 033-Civil engineering technicians | 4/11 | 0.48[0.14-1.61] | 0.63[0.29-1.37] | 0.59[0.23-1.53] | 0.64[0.28-1.49] | 0.71[0.36-1.40] | 0.81[0.53-1.25] |
| 034-Electrical and electronics engineering technicians | 5/9 | 1.61[0.44-5.88] | 1.00[0.45-2.21] | 1.21[0.45-3.22] | 1.12[0.47-2.66] | 1.02[0.51-2.02] | 0.93[0.60-1.44] |
| 035-Mechanical engineering technicians | 5/18 | 0.40[0.14-1.13] | 0.56[0.27-1.16] | 0.51[0.22-1.19] | 0.56[0.26-1.21] | 0.64[0.34-1.22] | 0.77[0.51-1.17] |
| 036-Chemical engineering technicians | 10/9 | 1.43[0.55-3.71] | 1.05[0.53-2.10] | 1.22[0.54-2.72] | 1.15[0.55-2.40] | 1.05[0.57-1.96] | 0.95[0.63-1.44] |
| 039-Engineering technicians not elsewhere classified | 7/7 | 1.71[0.51-5.72] | 1.05[0.49-2.28] | 1.28[0.50-3.28] | 1.18[0.51-2.72] | 1.05[0.54-2.07] | 0.95[0.62-1.46] |
| 061-Medical doctors | 4/9 | 0.46[0.13-1.58] | 0.63[0.29-1.36] | 0.58[0.22-1.51] | 0.63[0.27-1.47] | 0.71[0.36-1.40] | 0.81[0.52-1.24] |
| 121-Lawyers | 4/9 | 0.65[0.18-2.39] | 0.72[0.33-1.58] | 0.72[0.27-1.93] | 0.75[0.31-1.78] | 0.79[0.40-1.58] | 0.84[0.55-1.30] |
| 131-University and higher education teachers | 2/11 | 0.42[0.08-2.10] | 0.65[0.28-1.51] | 0.60[0.20-1.81] | 0.66[0.26-1.70] | 0.74[0.36-1.53] | 0.83[0.53-1.29] |
| 132-Secondary education teachers | 6/28 | 0.36[0.14-0.94] | 0.52[0.26-1.04] | 0.46[0.21-1.03] | 0.51[0.24-1.06] | 0.60[0.32-1.11] | 0.74[0.49-1.12] |
| 133-Primary education teachers | 5/16 | 0.50[0.17-1.48] | 0.63[0.30-1.31] | 0.59[0.25-1.44] | 0.64[0.29-1.41] | 0.71[0.37-1.35] | 0.80[0.53-1.23] |
| 159-Authors, journalists and related writers not elsewhere classified | 1/9 | 0.15[0.02-1.25] | 0.57[0.23-1.43] | 0.47[0.14-1.59] | 0.56[0.20-1.56] | 0.68[0.32-1.46] | 0.81[0.51-1.27] |
| 163-Photographers and cameramen | 6/4 | 1.19[0.32-4.37] | 0.89[0.41-1.96] | 1.01[0.38-2.71] | 0.97[0.41-2.32] | 0.93[0.47-1.86] | 0.90[0.58-1.39] |
| 211-General managers | 19/42 | 0.63[0.35-1.14] | 0.67[0.40-1.10] | 0.66[0.38-1.13] | 0.67[0.40-1.13] | 0.70[0.44-1.13] | 0.77[0.54-1.11] |
| 212-Production managers (except farm) | 4/13 | 0.41[0.13-1.33] | 0.59[0.28-1.26] | 0.54[0.21-1.35] | 0.59[0.26-1.35] | 0.67[0.35-1.32] | 0.79[0.52-1.21] |
| 219-Managers not elsewhere classified | 18/50 | 0.50[0.28-0.89] | 0.56[0.34-0.92] | 0.54[0.31-0.92] | 0.56[0.33-0.93] | 0.60[0.38-0.96] | 0.71[0.49-1.01] |
| 300-Clerical Supervisors | 3/13 | 0.30[0.08-1.16] | 0.55[0.25-1.24] | 0.47[0.17-1.29] | 0.54[0.22-1.30] | 0.65[0.32-1.30] | 0.78[0.51-1.21] |
| 310-Government Executive Officials | 7/8 | 1.55[0.53-4.53] | 1.06[0.51-2.20] | 1.25[0.52-2.99] | 1.17[0.53-2.57] | 1.06[0.55-2.02] | 0.95[0.62-1.45] |
| 331-Bookkeepers and cashiers | 31/48 | 0.91[0.55-1.49] | 0.87[0.56-1.36] | 0.90[0.56-1.44] | 0.89[0.57-1.41] | 0.89[0.58-1.36] | 0.89[0.63-1.24] |
| 339-Bookkeepers, cashiers and related workers not elsewhere classified | 20/53 | 0.48[0.28-0.84] | 0.54[0.33-0.88] | 0.52[0.31-0.87] | 0.53[0.32-0.88] | 0.58[0.37-0.92] | 0.69[0.48-0.98] |
| 359-Transport and communications supervisors not elsewhere classified | 1/16 | 0.08[0.01-0.65] | 0.51[0.20-1.28] | 0.37[0.11-1.25] | 0.48[0.17-1.32] | 0.62[0.29-1.33] | 0.78[0.50-1.23] |
| 360-Transport Conductors | 6/19 | 0.36[0.14-0.96] | 0.53[0.26-1.06] | 0.46[0.21-1.05] | 0.51[0.24-1.08] | 0.60[0.32-1.12] | 0.75[0.49-1.13] |
| **ISCO code - Occupation** | **Cases/**  **Controls** | **ML** | **SB** *7-fold range* | **HR** | | | |
|  |  |  |  | ***τ*=0.76** *20-fold range* | ***τ*=0.59** *10-fold range* | ***τ*=0.41** *5-fold range* | ***τ*=0.23** *2,5-fold range* |
|  |  | **OR[95%CI]** | **OR[95%CI]** | **OR[95%CI]** | **OR[95%CI]** | **OR[95%CI]** | **OR[95%CI]** |
| 370-Mail Distribution Clerks | 59/59 | 1.44[0.95-2.17] | 1.31[0.89-1.93] | 1.38[0.93-2.06] | 1.35[0.92-1.99] | 1.29[0.89-1.86] | 1.14[0.84-1.56] |
| 380-Telephone and Telegraph Operators | 18/22 | 1.01[0.51-1.99] | 0.92[0.53-1.62] | 0.98[0.53-1.81] | 0.96[0.54-1.73] | 0.94[0.56-1.59] | 0.91[0.62-1.34] |
| 391-Stock clerks | 43/65 | 0.79[0.51-1.20] | 0.78[0.53-1.16] | 0.79[0.52-1.19] | 0.79[0.53-1.18] | 0.80[0.55-1.17] | 0.82[0.60-1.13] |
| 392-Material and production planning clerks | 8/18 | 0.77[0.30-1.96] | 0.77[0.39-1.51] | 0.78[0.35-1.73] | 0.79[0.38-1.65] | 0.82[0.44-1.51] | 0.85[0.56-1.29] |
| 393-Correspondence and reporting clerks | 98/184 | 0.71[0.53-0.94] | 0.71[0.54-0.93] | 0.71[0.54-0.94] | 0.71[0.54-0.94] | 0.72[0.55-0.94] | 0.75[0.59-0.95] |
| 394-Receptionists and travel agency clerks | 3/8 | 0.39[0.10-1.56] | 0.61[0.27-1.37] | 0.55[0.20-1.53] | 0.61[0.25-1.49] | 0.70[0.35-1.41] | 0.81[0.52-1.25] |
| 395-Library and filing clerks | 6/9 | 1.04[0.33-3.27] | 0.87[0.41-1.84] | 0.95[0.38-2.37] | 0.93[0.41-2.11] | 0.91[0.47-1.77] | 0.89[0.58-1.37] |
| 399-Clerks not elsewhere classified | 5/9 | 0.82[0.24-2.82] | 0.79[0.36-1.69] | 0.82[0.32-2.13] | 0.83[0.36-1.93] | 0.84[0.43-1.66] | 0.87[0.56-1.33] |
| 410-Working Proprietors (Wholesale and Retail Trade) | 44/69 | 0.78[0.52-1.18] | 0.78[0.53-1.14] | 0.78[0.53-1.17] | 0.79[0.53-1.16] | 0.79[0.55-1.15] | 0.82[0.60-1.12] |
| 421-Sales supervisors | 5/10 | 0.50[0.16-1.51] | 0.63[0.30-1.32] | 0.59[0.24-1.45] | 0.64[0.28-1.43] | 0.71[0.37-1.36] | 0.80[0.53-1.23] |
| 431-Technical salesmen and service advisers | 9/14 | 0.75[0.31-1.81] | 0.76[0.39-1.46] | 0.77[0.36-1.64] | 0.78[0.39-1.58] | 0.80[0.44-1.46] | 0.84[0.56-1.27] |
| 432-Commercial travellers and Manufacturers' agents | 42/68 | 0.72[0.48-1.10] | 0.73[0.50-1.07] | 0.73[0.49-1.09] | 0.73[0.50-1.09] | 0.75[0.52-1.09] | 0.79[0.58-1.07] |
| 441-Insurance, real estate and securities salesmen | 7/16 | 0.57[0.23-1.45] | 0.66[0.33-1.29] | 0.63[0.29-1.39] | 0.66[0.32-1.37] | 0.72[0.39-1.32] | 0.80[0.53-1.21] |
| 451-Salesmen, shop assistants and demonstrators | 68/61 | 1.23[0.84-1.80] | 1.16[0.81-1.66] | 1.20[0.83-1.74] | 1.19[0.83-1.70] | 1.15[0.82-1.63] | 1.07[0.79-1.43] |
| 452-Street vendors, canvassers and newsvendors | 54/48 | 1.51[0.98-2.34] | 1.35[0.90-2.03] | 1.44[0.95-2.19] | 1.40[0.93-2.11] | 1.33[0.90-1.95] | 1.16[0.84-1.59] |
| 510-Working Proprietors (Catering and Lodging Services) | 18/20 | 1.29[0.64-2.61] | 1.08[0.61-1.93] | 1.19[0.63-2.25] | 1.15[0.63-2.10] | 1.08[0.64-1.84] | 0.98[0.67-1.44] |
| 531-Cooks | 24/25 | 0.95[0.52-1.72] | 0.90[0.54-1.49] | 0.93[0.54-1.62] | 0.92[0.54-1.57] | 0.91[0.57-1.48] | 0.90[0.62-1.29] |
| 532-Waiters, bartenders and related workers | 34/52 | 0.71[0.45-1.14] | 0.72[0.47-1.10] | 0.72[0.46-1.13] | 0.73[0.47-1.12] | 0.75[0.50-1.12] | 0.79[0.57-1.10] |
| 540-Maids and Related Housekeeping Service Workers Not Elsewhere Classified | 7/12 | 0.67[0.24-1.86] | 0.72[0.36-1.45] | 0.72[0.31-1.66] | 0.74[0.34-1.59] | 0.78[0.41-1.47] | 0.83[0.55-1.27] |
| 551-Building caretakers | 22/30 | 0.96[0.53-1.72] | 0.90[0.54-1.49] | 0.94[0.55-1.61] | 0.94[0.57-1.57] | 0.95[0.60-1.50] | 0.99[0.70-1.39] |
| 552-Charworkers, cleaners and related workers | 25/27 | 1.37[0.76-2.47] | 1.17[0.70-1.96] | 1.28[0.74-2.22] | 1.24[0.73-2.09] | 1.16[0.72-1.87] | 1.03[0.72-1.48] |
| 570-Hairdressers, Barbers, Beauticians and Related Workers | 16/22 | 0.95[0.47-1.91] | 0.88[0.50-1.56] | 0.93[0.49-1.74] | 0.92[0.51-1.67] | 0.91[0.53-1.54] | 0.89[0.61-1.31] |
| 582-Policemen and detectives | 37/43 | 1.18[0.73-1.90] | 1.08[0.70-1.67] | 1.14[0.72-1.80] | 1.12[0.72-1.74] | 1.08[0.72-1.63] | 1.00[0.72-1.40] |
| 589-Protective service workers not elsewhere classified | 19/19 | 1.52[0.78-2.99] | 1.22[0.69-2.15] | 1.37[0.74-2.54] | 1.31[0.73-2.35] | 1.20[0.71-2.01] | 1.04[0.71-1.51] |
| 599-Other service workers | 9/12 | 1.14[0.44-2.98] | 0.94[0.47-1.87] | 1.04[0.46-2.33] | 1.01[0.48-2.11] | 0.96[0.52-1.79] | 0.92[0.60-1.39] |
| 611-General farmers | 58/86 | 1.01[0.69-1.47] | 1.01[0.71-1.44] | 1.01[0.70-1.46] | 1.02[0.71-1.45] | 1.02[0.73-1.43] | 1.04[0.78-1.39] |
| **ISCO code - Occupation** | **Cases/**  **Controls** | **ML** | **SB** *7-fold range* | **HR** | | | |
|  |  |  |  | ***τ*=0.76** *20-fold range* | ***τ*=0.59** *10-fold range* | ***τ*=0.41** *5-fold range* | ***τ*=0.23** *2,5-fold range* |
|  |  | **OR[95%CI]** | **OR[95%CI]** | **OR[95%CI]** | **OR[95%CI]** | **OR[95%CI]** | **OR[95%CI]** |
| 612-Specialised farmers | 9/3 | 3.44[0.90-13.17] | 1.59[0.71-3.55] | 1.81[0.67-4.93] | 1.53[0.63-3.68] | 1.23[0.61-2.47] | 1.00[0.65-1.55] |
| 621-General farm workers | 146/222 | 0.90[0.70-1.16] | 0.91[0.71-1.16] | 0.91[0.71-1.16] | 0.91[0.71-1.16] | 0.92[0.73-1.17] | 0.95[0.76-1.18] |
| 622-Field crop and vegetable farm workers | 6/15 | 0.47[0.17-1.28] | 0.71[0.35-1.44] | 0.65[0.29-1.44] | 0.72[0.35-1.48] | 0.83[0.45-1.50] | 0.96[0.64-1.45] |
| 623-Orchard, vineyard and related tree and shrub crop workers | 5/7 | 1.01[0.29-3.58] | 1.04[0.48-2.25] | 0.93[0.35-2.44] | 0.91[0.39-2.15] | 0.89[0.45-1.77] | 0.89[0.57-1.37] |
| 624-Livestock workers | 6/16 | 0.51[0.19-1.39] | 0.74[0.37-1.49] | 0.59[0.26-1.36] | 0.63[0.30-1.35] | 0.70[0.37-1.31] | 0.80[0.52-1.21] |
| 627-Nursery workers and gardeners | 5/14 | 0.41[0.14-1.19] | 0.68[0.33-1.42] | 0.61[0.26-1.41] | 0.69[0.33-1.46] | 0.81[0.44-1.50] | 0.96[0.63-1.45] |
| 628-Farm machinery operators | 9/8 | 1.62[0.55-4.81] | 1.28[0.62-2.65] | 1.44[0.58-3.54] | 1.38[0.59-3.22] | 1.31[0.61-2.81] | 1.22[0.62-2.40] |
| 631-Loggers | 4/8 | 0.69[0.20-2.43] | 0.90[0.41-1.95] | 0.74[0.28-1.95] | 0.77[0.33-1.80] | 0.80[0.41-1.59] | 0.85[0.55-1.31] |
| 641-Fishermen | 8/7 | 1.51[0.51-4.51] | 1.24[0.59-2.57] | 1.22[0.50-2.96] | 1.14[0.51-2.54] | 1.04[0.54-2.00] | 0.94[0.62-1.44] |
| 700-Production Supervisors and General Foremen | 19/36 | 0.62[0.34-1.13] | 0.72[0.43-1.20] | 0.65[0.37-1.12] | 0.66[0.39-1.12] | 0.69[0.43-1.12] | 0.77[0.53-1.11] |
| 711-Miners and quarrymen | 17/17 | 1.19[0.58-2.44] | 1.14[0.63-2.03] | 1.25[0.64-2.45] | 1.27[0.66-2.45] | 1.30[0.69-2.47] | 1.34[0.73-2.49] |
| 721-Metal smelting, converting and refining furnacemen | 28/32 | 1.07[0.62-1.84] | 1.06[0.66-1.71] | 1.20[0.73-1.97] | 1.26[0.79-2.02] | 1.37[0.88-2.11] | 1.53[1.05-2.24] |
| 722-Metal rolling-mill workers | 6/9 | 0.78[0.26-2.29] | 0.92[0.44-1.90] | 0.85[0.37-1.96] | 0.87[0.41-1.84] | 0.90[0.49-1.66] | 0.93[0.62-1.40] |
| 723-Metal melters and reheaters | 12/6 | 2.06[0.75-5.72] | 1.45[0.71-2.96] | 1.84[0.87-3.90] | 1.79[0.91-3.50] | 1.73[0.99-3.03] | 1.70[1.11-2.61] |
| 724-Metal casters | 4/8 | 0.58[0.16-2.08] | 0.84[0.39-1.84] | 0.91[0.38-2.14] | 0.98[0.47-2.07] | 1.07[0.59-1.91] | 1.12[0.76-1.65] |
| 725-Metal moulders and coremakers | 10/9 | 1.72[0.65-4.58] | 1.35[0.67-2.70] | 1.48[0.70-3.13] | 1.41[0.72-2.76] | 1.32[0.77-2.28] | 1.21[0.83-1.77] |
| 726-Metal annealers, temperers and case-hardeners | 4/7 | 1.14[0.31-4.15] | 1.08[0.50-2.36] | 1.38[0.57-3.36] | 1.42[0.65-3.09] | 1.44[0.77-2.71] | 1.44[0.92-2.28] |
| 727-Metal drawers and extruders | 5/5 | 1.72[0.45-6.63] | 1.24[0.56-2.76] | 1.63[0.66-4.03] | 1.59[0.72-3.52] | 1.54[0.81-2.91] | 1.48[0.94-2.33] |
| 728-Metal platers and coaters | 13/7 | 3.26[1.17-9.07] | 1.81[0.88-3.72] | 2.30[1.05-5.05] | 2.08[1.02-4.23] | 1.82[1.00-3.29] | 1.57[1.01-2.46] |
| 729-Metal processers not elsewhere classified | 28/20 | 1.79[0.97-3.32] | 1.54[0.91-2.61] | 1.63[0.95-2.82] | 1.57[0.94-2.62] | 1.45[0.92-2.28] | 1.28[0.91-1.80] |
| 741-Crushers, grinders and mixers | 4/10 | 0.58[0.17-1.96] | 0.83[0.39-1.79] | 0.67[0.26-1.72] | 0.70[0.30-1.63] | 0.76[0.39-1.49] | 0.83[0.54-1.28] |
| 749-Chemical processers and related workers not elsewhere classified | 7/7 | 1.36[0.46-4.00] | 1.18[0.57-2.44] | 1.14[0.48-2.75] | 1.08[0.49-2.39] | 1.01[0.53-1.93] | 0.93[0.61-1.42] |
| 751-Fibre preparers | 5/5 | 1.97[0.48-8.03] | 1.29[0.58-2.89] | 1.31[0.47-3.64] | 1.18[0.48-2.90] | 1.05[0.52-2.12] | 0.94[0.61-1.46] |
| 761-Tanners and fellmongers | 5/8 | 1.12[0.34-3.64] | 1.08[0.51-2.29] | 0.99[0.39-2.51] | 0.96[0.42-2.21] | 0.93[0.48-1.81] | 0.90[0.59-1.38] |
| 771-Grain millers and related workers | 3/9 | 0.30[0.08-1.12] | 0.67[0.30-1.50] | 0.47[0.17-1.26] | 0.53[0.22-1.28] | 0.64[0.32-1.28] | 0.78[0.50-1.20] |
| 772-Sugar processers and refiners | 8/8 | 0.92[0.32-2.58] | 0.98[0.48-2.01] | 0.88[0.38-2.08] | 0.88[0.40-1.91] | 0.87[0.46-1.66] | 0.88[0.58-1.34] |
| 773-Butchers and meat preparers | 16/28 | 0.74[0.38-1.42] | 0.82[0.47-1.42] | 0.75[0.41-1.37] | 0.76[0.43-1.35] | 0.78[0.47-1.30] | 0.83[0.57-1.20] |
| **ISCO code - Occupation** | **Cases/**  **Controls** | **ML** | **SB** *7-fold range* | **HR** | | | |
|  |  |  |  | ***τ*=0.76** *20-fold range* | ***τ*=0.59** *10-fold range* | ***τ*=0.41** *5-fold range* | ***τ*=0.23** *2,5-fold range* |
|  |  | **OR[95%CI]** | **OR[95%CI]** | **OR[95%CI]** | **OR[95%CI]** | **OR[95%CI]** | **OR[95%CI]** |
| 776-Bakers, pastrycooks and confectionery makers | 51/54 | 1.19[0.78-1.82] | 1.17[0.79-1.73] | 1.16[0.77-1.74] | 1.14[0.77-1.70] | 1.11[0.76-1.61] | 1.03[0.75-1.41] |
| 791-Tailors and dressmakers | 14/11 | 2.08[0.87-5.00] | 1.54[0.79-2.97] | 1.64[0.77-3.50] | 1.49[0.74-3.01] | 1.28[0.71-2.32] | 1.05[0.70-1.57] |
| 796-Upholsterers and related workers | 19/11 | 2.27[0.99-5.21] | 1.65[0.87-3.13] | 1.79[0.87-3.69] | 1.62[0.82-3.18] | 1.37[0.77-2.45] | 1.08[0.72-1.62] |
| 801-Shoemakers and shoe repairers | 29/32 | 1.28[0.73-2.23] | 1.22[0.75-1.98] | 1.21[0.72-2.04] | 1.18[0.71-1.95] | 1.12[0.71-1.77] | 1.01[0.71-1.45] |
| 802-Shoe cutters, lasters, sewers and related workers | 7/7 | 1.14[0.37-3.52] | 1.09[0.52-2.28] | 1.01[0.41-2.50] | 0.98[0.44-2.20] | 0.94[0.49-1.82] | 0.90[0.59-1.39] |
| 811-Cabinetmakers | 23/30 | 1.09[0.59-2.02] | 1.08[0.64-1.82] | 1.05[0.59-1.85] | 1.03[0.60-1.77] | 0.99[0.61-1.63] | 0.94[0.65-1.36] |
| 812-Woodworking-machine operators | 7/7 | 1.34[0.43-4.18] | 1.16[0.55-2.45] | 1.12[0.45-2.77] | 1.06[0.47-2.39] | 0.99[0.51-1.92] | 0.92[0.60-1.41] |
| 819-Cabinetmakers and related woodworkers not elsewhere classified | 13/22 | 1.06[0.48-2.31] | 1.05[0.57-1.94] | 1.00[0.50-2.00] | 0.98[0.51-1.88] | 0.95[0.54-1.67] | 0.91[0.61-1.36] |
| 831-Blacksmiths, hammersmiths and forging-press operators | 24/33 | 1.02[0.57-1.81] | 1.03[0.62-1.69] | 1.02[0.61-1.71] | 1.02[0.63-1.67] | 1.04[0.67-1.60] | 1.07[0.77-1.49] |
| 832-Toolmakers, metal patternmakers and metal markers | 6/25 | 0.29[0.11-0.74] | 0.54[0.27-1.08] | 0.50[0.24-1.04] | 0.59[0.31-1.14] | 0.74[0.43-1.27] | 0.95[0.66-1.37] |
| 833-Machine-tool setter-operators | 11/12 | 1.18[0.48-2.91] | 1.12[0.58-2.18] | 1.10[0.52-2.32] | 1.07[0.54-2.12] | 1.03[0.58-1.83] | 0.98[0.66-1.46] |
| 834-Machine-tool operators | 75/114 | 0.90[0.64-1.26] | 0.92[0.67-1.26] | 0.91[0.65-1.26] | 0.91[0.66-1.25] | 0.91[0.67-1.24] | 0.92[0.71-1.21] |
| 835-Metal grinders, polishers and tool sharpeners | 7/13 | 0.59[0.23-1.56] | 0.79[0.39-1.57] | 0.76[0.36-1.60] | 0.82[0.42-1.59] | 0.91[0.53-1.56] | 1.03[0.71-1.49] |
| 839-Blacksmiths, toolmakers and machine-tool operators not elsewhere classified | 34/26 | 1.51[0.86-2.65] | 1.38[0.85-2.25] | 1.40[0.83-2.37] | 1.35[0.82-2.24] | 1.25[0.79-1.99] | 1.08[0.76-1.54] |
| 841-Machinery fitters and machine assemblers | 57/62 | 1.35[0.90-2.03] | 1.30[0.89-1.89] | 1.29[0.88-1.91] | 1.27[0.87-1.85] | 1.22[0.86-1.75] | 1.15[0.86-1.54] |
| 842-Watch, clock and precision instrument makers | 6/7 | 0.78[0.25-2.42] | 0.93[0.44-1.94] | 0.80[0.32-1.97] | 0.81[0.36-1.82] | 0.83[0.43-1.60] | 0.86[0.56-1.31] |
| 843-Motor vehicle mechanics | 27/35 | 0.87[0.51-1.50] | 0.91[0.57-1.46] | 0.87[0.53-1.44] | 0.88[0.54-1.42] | 0.90[0.58-1.38] | 0.95[0.68-1.33] |
| 849-Machinery fitters, machine assemblers and precision instrument makers (except electrical) not elsewhere classified | 89/115 | 0.96[0.70-1.31] | 0.97[0.72-1.31] | 0.97[0.71-1.31] | 0.97[0.72-1.30] | 0.98[0.74-1.30] | 1.02[0.80-1.31] |
| 851-Electrical fitters | 11/26 | 0.54[0.25-1.14] | 0.69[0.38-1.26] | 0.61[0.32-1.18] | 0.65[0.35-1.20] | 0.73[0.43-1.23] | 0.88[0.61-1.27] |
| 852-Electronics fitters | 7/8 | 1.00[0.34-2.94] | 1.03[0.50-2.12] | 0.94[0.39-2.25] | 0.92[0.42-2.03] | 0.90[0.47-1.73] | 0.89[0.58-1.36] |
| 853-Electrical and electronics equipment assemblers | 4/6 | 1.08[0.27-4.24] | 1.06[0.48-2.35] | 0.95[0.35-2.62] | 0.93[0.38-2.26] | 0.90[0.45-1.82] | 0.89[0.57-1.38] |
| 855-Electrical wiremen | 30/33 | 1.34[0.78-2.31] | 1.26[0.78-2.04] | 1.25[0.76-2.07] | 1.22[0.75-1.97] | 1.17[0.75-1.80] | 1.10[0.79-1.53] |
| **ISCO code - Occupation** | **Cases/**  **Controls** | **ML** | **SB** *7-fold range* | **HR** | | | |
|  |  |  |  | ***τ*=0.76** *20-fold range* | ***τ*=0.59** *10-fold range* | ***τ*=0.41** *5-fold range* | ***τ*=0.23** *2,5-fold range* |
|  |  | **OR[95%CI]** | **OR[95%CI]** | **OR[95%CI]** | **OR[95%CI]** | **OR[95%CI]** | **OR[95%CI]** |
| 856-Telephone and telegraph installers | 3/10 | 0.29[0.08-1.10] | 0.67[0.30-1.50] | 0.46[0.17-1.25] | 0.53[0.22-1.27] | 0.64[0.32-1.28] | 0.78[0.50-1.20] |
| 857-Electric linemen and cable jointers | 6/10 | 0.57[0.20-1.62] | 0.79[0.38-1.62] | 0.64[0.27-1.52] | 0.68[0.31-1.48] | 0.73[0.39-1.39] | 0.81[0.53-1.24] |
| 871-Plumbers and pipe fitters | 29/22 | 1.94[1.03-3.65] | 1.62[0.95-2.77] | 1.90[1.06-3.43] | 1.87[1.05-3.32] | 1.82[1.05-3.16] | 1.76[1.04-2.98] |
| 872-Welders and flame-cutters | 47/37 | 1.67[1.03-2.71] | 1.53[0.99-2.36] | 1.66[1.06-2.59] | 1.64[1.06-2.53] | 1.60[1.07-2.41] | 1.53[1.06-2.19] |
| 873-Sheet-metal workers | 16/35 | 0.62[0.33-1.16] | 0.72[0.42-1.23] | 0.67[0.38-1.19] | 0.70[0.41-1.21] | 0.76[0.47-1.22] | 0.85[0.59-1.22] |
| 874-Structural metal preparers and erectors | 18/23 | 1.15[0.59-2.23] | 1.12[0.64-1.94] | 1.10[0.61-2.00] | 1.09[0.62-1.90] | 1.05[0.64-1.72] | 1.00[0.69-1.44] |
| 880-Jewellery and Precious Metal Workers | 3/8 | 0.29[0.07-1.13] | 0.68[0.30-1.52] | 0.47[0.17-1.28] | 0.54[0.22-1.30] | 0.64[0.32-1.29] | 0.78[0.50-1.21] |
| 891-Glass formers, cutters, grinders and finishers | 15/8 | 1.95[0.78-4.85] | 1.46[0.75-2.87] | 1.53[0.72-3.24] | 1.40[0.71-2.78] | 1.24[0.70-2.19] | 1.06[0.71-1.57] |
| 893-Glass and ceramics kilnmen | 11/6 | 2.14[0.73-6.25] | 1.45[0.70-3.01] | 1.61[0.76-3.42] | 1.53[0.79-2.95] | 1.44[0.85-2.45] | 1.38[0.95-2.00] |
| 901-Rubber and plastics product makers (except tire makers and tire vulcanisers) | 22/27 | 1.10[0.59-2.04] | 1.08[0.64-1.83] | 1.05[0.59-1.87] | 1.03[0.60-1.79] | 1.00[0.61-1.64] | 0.94[0.65-1.37] |
| 902-Tire makers and vulcanisers | 6/10 | 0.81[0.28-2.39] | 0.93[0.45-1.93] | 0.82[0.34-1.96] | 0.82[0.37-1.82] | 0.84[0.44-1.60] | 0.86[0.56-1.32] |
| 921-Compositors and typesetters | 8/11 | 1.14[0.40-3.23] | 1.09[0.54-2.23] | 1.03[0.44-2.41] | 0.99[0.46-2.16] | 0.95[0.50-1.80] | 0.91[0.60-1.39] |
| 931-Painters, construction | 42/29 | 1.85[1.09-3.15] | 1.63[1.02-2.61] | 1.66[1.01-2.71] | 1.57[0.98-2.52] | 1.43[0.93-2.20] | 1.23[0.89-1.72] |
| 939-Painters not elsewhere classified | 22/15 | 1.58[0.77-3.22] | 1.37[0.77-2.45] | 1.40[0.73-2.66] | 1.32[0.72-2.43] | 1.20[0.70-2.06] | 1.03[0.70-1.52] |
| 943-Non-metallic mineral product makers | 7/4 | 2.00[0.56-7.13] | 1.34[0.61-2.92] | 1.51[0.64-3.56] | 1.42[0.68-2.98] | 1.31[0.73-2.35] | 1.21[0.82-1.78] |
| 949-Other production and related workers | 3/19 | 0.22[0.06-0.78] | 0.58[0.26-1.29] | 0.38[0.15-1.00] | 0.45[0.19-1.07] | 0.58[0.29-1.14] | 0.75[0.48-1.15] |
| 951-Bricklayers, stonemasons and tile setters | 77/75 | 1.34[0.94-1.92] | 1.31[0.94-1.82] | 1.33[0.95-1.87] | 1.33[0.95-1.84] | 1.30[0.95-1.78] | 1.24[0.95-1.63] |
| 952-Reinforced-concreters, cement finishers and terrazzo workers | 13/8 | 1.78[0.70-4.56] | 1.38[0.70-2.74] | 1.52[0.73-3.16] | 1.44[0.75-2.78] | 1.34[0.78-2.30] | 1.22[0.84-1.77] |
| 954-Carpenters, joiners and parquetry workers | 28/49 | 0.81[0.49-1.35] | 0.86[0.54-1.35] | 0.81[0.50-1.32] | 0.82[0.51-1.30] | 0.82[0.53-1.27] | 0.84[0.60-1.19] |
| 959-Construction workers not elsewhere classified | 19/19 | 1.24[0.63-2.47] | 1.18[0.67-2.07] | 1.21[0.67-2.21] | 1.21[0.69-2.12] | 1.21[0.74-1.98] | 1.23[0.84-1.79] |
| 969-Stationary engine and related equipment operators not elsewhere classified | 14/18 | 0.80[0.38-1.69] | 0.88[0.49-1.60] | 0.81[0.41-1.57] | 0.81[0.43-1.52] | 0.82[0.48-1.43] | 0.85[0.58-1.26] |
| 971-Dockers and freight handlers | 93/91 | 1.24[0.89-1.71] | 1.22[0.89-1.66] | 1.21[0.88-1.66] | 1.20[0.88-1.63] | 1.18[0.87-1.58] | 1.13[0.87-1.46] |
| 973-Crane and hoist operators | 11/14 | 1.14[0.48-2.67] | 1.10[0.58-2.09] | 1.05[0.50-2.21] | 1.02[0.51-2.03] | 0.98[0.54-1.76] | 0.92[0.62-1.39] |
| 974-Earth-moving and related machinery operators | 13/16 | 0.97[0.45-2.12] | 1.00[0.54-1.84] | 1.00[0.51-1.96] | 1.01[0.54-1.89] | 1.03[0.60-1.76] | 1.05[0.71-1.55] |
| 979-Material-handling equipment operators not elsewhere classified | 10/23 | 0.63[0.28-1.40] | 0.77[0.41-1.44] | 0.67[0.33-1.36] | 0.69[0.36-1.34] | 0.73[0.41-1.29] | 0.81[0.54-1.20] |

| **ISCO code - Occupation** | **Cases/**  **Controls** | **ML** | **SB** *7-fold range* | **HR** | | | |
| --- | --- | --- | --- | --- | --- | --- | --- |
|  |  |  |  | ***τ*=0.76** *20-fold range* | ***τ*=0.59** *10-fold range* | ***τ*=0.41** *5-fold range* | ***τ*=0.23** *2,5-fold range* |
|  |  | **OR[95%CI]** | **OR[95%CI]** | **OR[95%CI]** | **OR[95%CI]** | **OR[95%CI]** | **OR[95%CI]** |
| 981-Ships' deck ratings, barge crews and boatmen | 9/11 | 0.74[0.30-1.88] | 0.88[0.45-1.72] | 0.79[0.37-1.68] | 0.81[0.41-1.62] | 0.87[0.49-1.53] | 0.96[0.65-1.40] |
| 982-Ships' engine-room ratings | 8/2 | 5.88[0.94-36.71] | 1.54[0.64-3.73] | 2.43[0.79-7.46] | 2.16[0.82-5.73] | 1.93[0.87-4.29] | 1.78[0.96-3.32] |
| 983-Railway engine drivers and firemen | 9/11 | 0.97[0.38-2.46] | 1.01[0.51-1.98] | 1.27[0.57-2.80] | 1.35[0.64-2.85] | 1.47[0.75-2.89] | 1.62[0.91-2.88] |
| 985-Motor vehicle drivers | 94/138 | 0.77[0.57-1.04] | 0.79[0.59-1.05] | 0.77[0.58-1.04] | 0.78[0.58-1.04] | 0.78[0.59-1.03] | 0.80[0.62-1.03] |
| 989-Transport equipment operators not elsewhere classified | 7/9 | 0.83[0.29-2.34] | 0.94[0.46-1.92] | 0.83[0.35-1.94] | 0.83[0.38-1.81] | 0.84[0.45-1.60] | 0.86[0.57-1.32] |
| 999-Labourers not elsewhere classified | 204/205 | 1.28[1.01-1.61] | 1.26[1.01-1.59] | 1.26[1.00-1.59] | 1.25[1.00-1.57] | 1.24[0.99-1.54] | 1.20[0.98-1.47] |
